# Current status and future directions for the development of human papillomavirus vaccines

**DOI:** 10.3389/fimmu.2024.1362770

**Published:** 2024-06-25

**Authors:** Rui Wang, Hongpeng Huang, Chulin Yu, Xuefeng Li, Yang Wang, Liangzhi Xie

**Affiliations:** ^1^ Beijing Engineering Research Center of Protein and Antibody, Sinocelltech Ltd., Beijing, China; ^2^ Cell Culture Engineering Center, Chinese Academy of Medical Sciences & Peking Union Medical College, Beijing, China

**Keywords:** human papillomavirus vaccine, prophylactic, therapeutic, antigens, adjuvants, formulation strategy

## Abstract

The development of human papillomavirus (HPV) vaccines has made substantive progress, as represented by the approval of five prophylactic vaccines since 2006. Generally, the deployment of prophylactic HPV vaccines is effective in preventing newly acquired infections and incidences of HPV-related malignancies. However, there is still a long way to go regarding the prevention of all HPV infections and the eradication of established HPV infections, as well as the subsequent progression to cancer. Optimizing prophylactic HPV vaccines by incorporating L1 proteins from more HPV subtypes, exploring adjuvants that reinforce cellular immune responses to eradicate HPV-infected cells, and developing therapeutic HPV vaccines used either alone or in combination with other cancer therapeutic modalities might bring about a new era getting closer to the vision to get rid of HPV infection and related diseases. Herein, we summarize strategies for the development of HPV vaccines, both prophylactic and therapeutic, with an emphasis on the selection of antigens and adjuvants, as well as implications for vaccine efficacy based on preclinical studies and clinical trials. Additionally, we outline current cutting-edge insights on formulation strategies, dosing schedules, and age expansion among HPV vaccine recipients, which might play important roles in addressing barriers to vaccine uptake, such as vaccine hesitancy and vaccine availability.

## 1 Introduction

Human papillomavirus (HPV) infection can cause multiple types of clinical manifestations or diseases such as genital warts, respiratory papillomatosis, head and neck cancer (mostly oropharyngeal squamous cell carcinoma), anal cancer, penile cancer, vulvar cancer, vaginal cancer, and cervical cancer (1, 2). Among these HPV-associated cancers, cervical cancer contributes to the highest proportion and ranks as the fourth most commonly diagnosed cancer and the fourth leading cause of cancer death in women (3, 4).

Despite the high efficacy demonstrated by prophylactic HPV vaccines and the benefit of cervical screening across the female population, the number of newly diagnosed cervical cancer patients in 2018 was nearly 570,000 cases, of which 311,000 cases died from this disease worldwide (5). Globally, in 2020, the estimated new cancer cases would extend to 660,000, with about 350,000 deaths (6).

Addressing barriers to vaccine uptake, such as vaccine hesitancy and vaccine availability, might be an effective approach for improving HPV vaccine coverage (7, 8). Furthermore, efforts to improve the efficacy of HPV vaccines, both prophylactic and therapeutic, could also play important roles in tackling HPV infection and the incidence of HPV-related cancers or genital warts. In this review, we summarize the status and prospects for the development of prophylactic and therapeutic HPV vaccines. Exploration of the dosing schedules, formulation strategies, and age expansion of vaccine recipients will also be discussed.

## 2 Characteristics of human papillomavirus

### 2.1 Classification of HPV

HPV is an essential member of the *Papillomaviridae* family, encompassing a wide range of primitive DNA viruses distributed among various host species (9). Phylogenetically, HPVs are categorized into five genera: *Alpha, Beta, Gamma, Mu, and Nu*, based on the homology of the nucleotide sequence in the L1 gene (9, 10). *Alpha* and *Beta* HPVs are the most commonly studied and have distinctive biological properties. *Beta* viruses frequently infect cutaneous epithelium and are involved in non-melanoma skin cancers, whereas *Alpha* genera-HPVs infect both mucosal and genital epithelium and are the causes of many anogenital cancers in humans and primates (11, 12). The *Alpha* HPVs are further grouped into low-risk HPVs (LR-HPVs) and high-risk HPVs (HR-HPVs) depending on their capacity to cause cancer. The LR-HPVs include HPV-6/11/42/43/44. HPV 6 and 11 cause most cases of genital warts (13). HPV-16/18/31/33/35/39/45/51/52/56/58/59/68 are members of HR-HPVs (14, 15).

### 2.2 Genomic characterization of HPV

**Figure 1:**
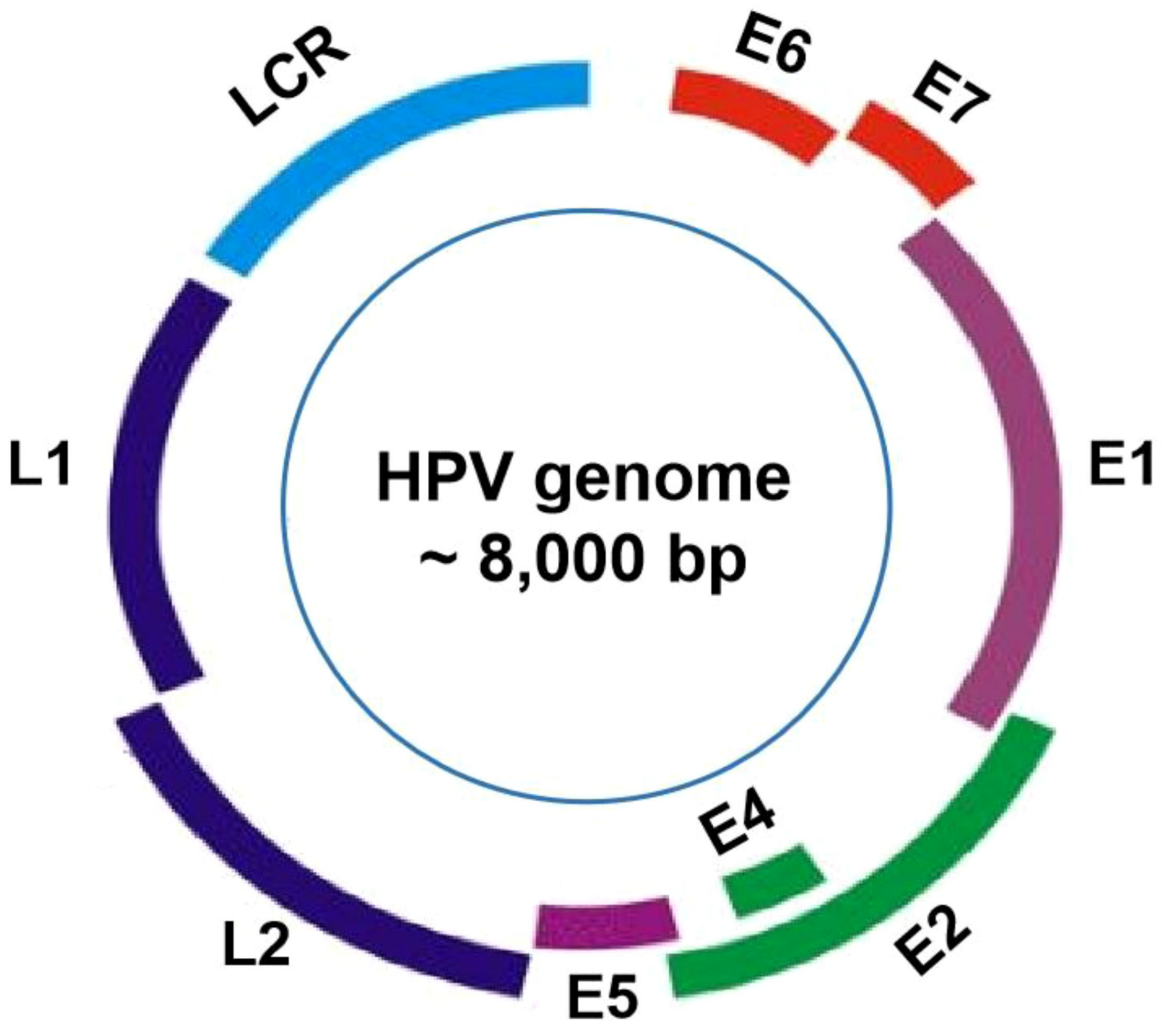
HPV genome organization. The genome of HPVs, about 8000 base pairs in length, is consists of approximately eight open reading frames (ORFs), which can be functionally categorized into three main regions: the E region, the L region, and the long control region (LCR).

The HPV viral particles are composed of a single double-stranded DNA molecule, with approximately 8,000 base pairs (bp), and a protein capsid containing 72 pentameric capsomers (16). The genomes of all HPV types contain about eight open-reading frames (ORFs) that can be functionally divided into the early (E), the late (L), and the long control region (LCR) (**Figure 1**). Specifically, the E gene encodes the early proteins (E1–E7) responsible for viral replication and oncogenesis. The L gene encodes the structural proteins (L1–L2) necessary for virion assembly. The non-coding region is thought to be the most highly variable part of the viral genome (17). The structural capsid proteins of HPVs are composed of two key proteins, major basic L1 and minor basic L2, respectively encoded by L1 and L2 genes. L1 genomes display differently amongst HPV types, while the L2 genomic sequence is relatively conserved (18). The HPV capsid is made up of 360 copies of major (L1) and 72 copies of minor (L2) capsid proteins (19). The L1 protein is self-assembled into the virus-like particles (VLP), while the L2 protein, as a minor structural molecule, is unable to form VLPs. Although the L2 protein is not responsible for binding to and entering cells during the process of infection, it plays a key role in enhancing L1 assembly into VLPs and viral genome encapsidation (20). Once entering host cells, the viral E6 and E7 oncoproteins are responsible for viral oncogenesis by affecting the function of tumor suppressors p53 and pRB (21). HPV types that cause genital warts are called low-risk, while those types that cause cervical cancer are considered high-risk (22). High-risk HPV genomes are commonly integrated into the host genome in most progressive cancer cases. Conversely, low-risk HPV genomes are commonly identified extra-chromosomally in benign and low-grade lesions, which are rarely found in tumors (23).

## 3 Current status of HPV vaccines

### 3.1 Currently available prophylactic HPV vaccines

Currently, five commercial HPV vaccines targeting high-risk HPV types have been approved for prophylactic use. All licensed vaccines contain L1 VLP. One of the primary distinctions among these authorized HPV vaccines is their level of valency.

Cervarix^®^ was licensed by the European Medicines Agency (EMA) in 2007 and by the U.S. Food and Drug Administration (FDA) in 2009. Cervarix^®^ is effective in defending against HPV-16 and HPV-18, responsible for nearly 70% of cervical cancers (24). Cervarix^®^ is formulated in a proprietary AS04 adjuvant containing aluminum hydroxide combined with a Toll-like receptor 4 (TLR4) ligand, 3-O-desacyl-4’-monophosphoryl lipid A (MPL) (25, 26). It is indicated for use in females aged 10 through 25 years for the prevention of persistent infection, premalignant cervical lesions, and cervical cancer caused by HPVs in the US.

Gardasil^®^, a quadrivalent HPV vaccine, is the first commercially available HPV vaccine approved in 2006 for people 9 through 26 years of age for the prevention of diseases caused by HPV infection (27). Aside from HPV-16 and 18, Gardasil^®^ also protects against HPV-6 and -11 infections, which cause approximately 90% of genital warts. Gardasil^®^ is adsorbed on an amorphous aluminum hydroxyphosphate sulfate adjuvant (28).

Gardasil 9^®^, licensed by the FDA in 2014, offers broader protection by covering five additional HPV types (HPV-31, 33, 45, 53, and 58) that might account for about 20% of cervical cancer cases (29). Gardasil 9^®^ is indicated for females aged 9 through 45 years for the prevention of HPV-associated diseases. Additionally, Gardasil 9^®^ is indicated in males 9 through 45 years of age for the prevention of anal, oropharyngeal, head neck cancers, anal precancerous, dysplastic lesions, and genital warts caused by HR-HPVs (30). Gardasil 9^®^ is formulated with amorphous aluminum hydroxyphosphate sulfate (AAHS) as an adjuvant (31).

Recently, two more bivalent HPV vaccines (HPV-16 and 18) have been approved in China for protection against HPV infection-induced precancerous lesions or high-grade genital lesions (32–34). Clinical data revealed that the 2-valent HPV vaccine showed favorable immunogenicity, safety, and a positive seroconvertsion rate (35–37). Both bivalent vaccines contain twice the amount of HPV-16 L1 protein as the Cervarix^®^ vaccine and use different adjuvants and antigen expression systems. A comprehensive comparison of marketed HPV vaccines is shown in **Table 1**.

**Table 1:** Summary of marketed prophylactic HPV vaccines.

Valency (Brand name)	2vHPV (Cervarix^®^)	4vHPV (Gardasil^®^)	9vHPV (Gardasil 9^®^)	2vHPV (Cecolin^®^)	2vHPV (WalrinVax^®^)
Approval	2009	2006	2014	2019	2022
Manufacturer	GlaxoSmithKline plc	Merck & Co., Inc	Merck & Co., Inc	Xiamen Innovax Co., Ltd.	Walvax Co., Ltd
Vaccine antigens (L1 protein)	HPV-16 (20 μg) HPV-18 (20 μg)	HPV-6,18 (20 μg each) HPV-11,16 (40 μg each)	HPV-6 (30 μg) HPV-16 (60 μg) HPV-11, 18 (40 μg each) HPV-31, 33, 45, 52, 58 (20 μg each)	HPV-16 (40 μg) HPV-18 (20 μg)	HPV-16 (40 μg) HPV-18 (20 μg)
Expression system	*Trichoplusiani* insect cell line	*Saccharomyces cerevisiae*	*Saccharomyces cerevisiae*	*Escherichia coli*	*Pichia pastoris*
Adjuvant	AS04	AAHS	AAHS	Aluminum hydroxide	Aluminum Phosphate
Other components	Sodium chloride and sodium dihydrogen phosphate dehydrate	Sodium chloride, L-histidine, polysorbate 80, sodium borate	Sodium chloride, L-histidine, polysorbate 80, sodium borate, yeast protein	Sodium chloride, sodium dihydrogen phosphate dehydrate, disodium hydrogen phosphate dehydrate, and polysorbate 80	Sodium chloride, histidine, and polysorbate 80
Volume per dose	0.5 mL	0.5 mL	0.5 mL	0.5 mL	0.5 mL
Route of administration	Intramuscular	Intramuscular	Intramuscular	Intramuscular	Intramuscular
Vaccination schedule	3 doses (0, 1–2, and 6 months apart)	3 doses (0, 1–2, and 6 months apart)	3 doses (0, 1–2, and 6 months apart)	3 doses (0, 1, and 6 months apart)	3 doses (0, 2, and 6 months apart)
Vaccine recipients	Females aged 10–25	Female and male aged 9–26	Female and male aged 9–45	Females aged 9–45	Female aged 9–30
Efficacy *	VE against HPV-16/18 incident infection was 66.8% (40)	100% efficacy against HPV 16/18-related cervical intraepithelial neoplasia and efficacy against HPV persistent infection for 78 months (41)	At 1 month post-Dose 3, >99% of participants in the per-protocol immunogenicity population seroconverted to each vaccine HPV type (42)	Vaccine efficacy was 100% against high-grade genital lesions and 97.3% against persistent infection (43)	100% in postive seropositive at Month 7 in two- and three-dose regimens for all participants (34)

*, Protection rate for precancerous lesions or high-grade genital lesions caused by HPV infection.

The immunogenicity and efficacy of five licensed HPV vaccines have been summarized elsewhere (33, 38, 39). Briefly, all marketed HPV vaccines elicit potent humoral immune responses and exhibited long-lasting protective efficacy after vaccination (34, 40–43). Serum antibody responses are the major indicators used for evaluating immunogenicity evoked by HPV vaccines in clinical trials, and comparison of vaccine-induced antibody levels or B-cell responses between HPV vaccines have been discussed (44–46). Nevertheless, definitive correlation between humoral immune responses and efficacy of HPV vaccines has not yet been reported (47). Prophylactic HPV vaccines based on L1-VLPs trigger T-cell responses, which showed no correlation with protection, probably due to the lack of L1 expression in HPV-infected cells (48). Since the protection efficacy of HPV vaccines are characterized in clinical trials by prevention of cervical cancer and intraepithelial neoplasia, immune profiles other than serum neutralizing antibody titers should be clarified, such as characteristics of Fc-effector functions, mucosal immunity and T-cell responses against HPV early proteins. For example, a comparative study between Gardasil^®^ and Cervarix^®^ indicated that the differences in antibody Fc-effector functions might contribute to post-infection protection (49). Immune correlate of protection by HPV vaccination needs further elucidation based on more clinical data with functional characteristic of serum or mucosal antibodies, as well as cellular immune responses (50).

Despite the high efficacy against HPV infection and HPV-related disease, low vaccination coverage of prophylactic HPV vaccines still exists. Particularly, most low- and middle-income countries incompletely implement HPV vaccination programs (51). The high cost, limited supply, and delivery requirements of the cold chain make it unavailable to people in these countries (52). Additionally, the biosafety considerations played a critical role in the successful development and deployment of HPV vaccines. Based on high quality data from very large studies, HPV vaccines exhibit favorable safety profiles (53, 54). The implementation of a robust safety monitoring system for HPV vaccines, which consistently updated data and promptly disclosed findings, would enhance accurate understanding and comprehensive awareness regarding HPV vaccine safety, mitigate vaccine hesitancy, and increase vaccine acceptance and coverage rates.

### 3.2 Prophylactic HPV vaccines in clinical testing

There are 12 high-risk HPV types that are involved in cancer, including types 16, 18, 31, 33, 35, 39, 45, 51, 52, 56, 58, and 59 (55). The Gardasil 9^®^ vaccine, which contains the highest number of high-risk HPV serotypes in circulation worldwide, does not fully cover all high-risk HPV types. To achieve complete protection, the development of prophylactic HPV vaccines should further enlarge their protective extent to target more life-threatening HPV types. The following section will summarize the progress in the development of prophylactic L1-VLP-based HPV vaccines currently in clinical trials (**Table 2**).

**Table 2:** Summary of prophylactic HPV vaccines in clinical trials.

HPV types	Expression System	Adjuvant	Sponsor	Indications	Efficacy and outcomes	Phase	ClinicalTrials.gov Identifier	Ref.
HPV-6/11/16/18/31/33/45/52/58 (9-valent)	N. A.	N. A.	Instituto de Investigación Hospital Universitario La Paz	HPV infection; HPV-Related Carcinoma.	N.A.	IV	NCT05439083	N.A.
HPV-6/11/16/18/31/33/45/52/58 (9-valent)	N. A.	N. A.	Shanghai Bovax Biotechnology Co., Ltd.	HPV Infections; HPV-Related Carcinoma.	Non-inferior to Gardasil 4 in immunogenicity and safety	III	NCT04895020	(56)
HPV-6/11/16/18/31/33/45/52/58 (9-valent)	N. A.	N. A.	Weill Medical College of Cornell University	HPV Positive Oropharyngeal Squamous Cell Carcinoma; HIV-1-infection; HPV Infection.	N.A.	III	NCT04255849	N.A.
HPV-6/11/16/18/31/33/45/52/58/59/68 (11-valent)	*Hansenulapolymorpha*	N. A.	National Vaccine and Serum Institute, China	HPV infection; HPV-Related Carcinoma.	N.A.	III	NCT05262010	N.A.
HPV-6/11/16/18/31/33/35/39/45/51/52/56/58/59 (14-valent, named SCT1000)	Insect cells	Aluminum-phosphate-based adjuvant	Sinocelltech Ltd.	HPV infection; HPV-Related Carcinoma.	N.A.	III	NCT06041061	N.A.
HPV-6/11/16/18/31/33/35/39/45/51/52/56/58/59/68 (15-valent)	*Escherichia coli*	N. A.	Liaoning Cheng Da Biotechnology Co., Ltd; Beijing Health Guard Biotechnology Inc.	HPV infection; HPV-Related Carcinoma.	N.A.	IND Approval	/	N.A.

N.A., not available.

All 9-valent HPV vaccines target the same highly carcinogenic HPV types (HPV-6/11/16/18/31/33/45/52/58). Shanghai Bovax Biotechnology Co., Ltd. has created a 9-valent HPV vaccine that exhibits high immunogenicity, good tolerance, and non-inferiority to Gardasil 4 in terms of both immunogenicity and safety (56). However, data regarding other HPV vaccines has not been made public. The 11-valent HPV vaccine, developed by the National Vaccine and Serum Institute (China), covered two additional HPV types (HPV-59/68) on top of the commonly used HPV types in 9-valent vaccines. The underlying development strategy might be linked to the findings that HPV-59 and 68 are potentially associated with genital cancerogenesis (57). SCT1000, developed by Sinocelltech Ltd, is a recombinant 14-valent L1-VLP-based HPV vaccine designed to prevent HPV infection and HPV-related cancers. It covers five additional HPV types (35, 39, 51, 56, and 59) compared with Gardasil 9^®^. SCT1000 is currently being investigated in a Phase III study for HPV infection and HPV-related carcinoma (NCT06041061). A 15-valent vaccine developed by Chengda Bio targets HPV-68, in addition to the 14 HPV types covered by SCT1000, is currently in development for the prevention of HPV-infected diseases.

### 3.3 Therapeutic HPV vaccines in clinical testing

Prophylactic HPV vaccines scarcely eradicate preexisting infection because L1 capsid proteins are not expressed on the surface of infected basal epithelial cells (58). Therefore, the development of prophylactic HPV vaccines is not an effective treatment approach for people already infected with HPV (59). Cellular immune responses to HPV antigens play important roles in viral clearance and anticancer immune responses. Ineffective cellular immune responses to HPV-16 E2, E6, or E7 peptides involve infection persistence or disease progression in low-grade intraepithelial lesion (LSIL) patients (60). HPV-16 E6 and E7-specific cellular immune responses facilitate the regression of HPV-16-associated lesions (61). Similarly, the percentages of IFN-γ positive enzyme-linked immunospot (ELISpot) responses to HPV-16 E6 and E7 are significantly increased among women with recently resolved HPV infection versus those with persistent HPV-16 infection in the cervix (62).

Therapeutic HPV vaccines are developed to stimulate cell-mediated immunity for the clearance of infection (63, 64). Among HPV early proteins (E1, E2, E6, and E7) and late proteins (L1, L2), HPV E6 and E7 proteins are constitutively expressed in both premalignant and invasive lesions and play key roles in the development of malignancy (65). Further, E6 and E7 can activate antigen-specific CD8^+^ or CD4^+^ T cells and finally evoke cellular immune responses (66–68). Therefore, E6 and E7 proteins are considered ideal antigens for therapeutic HPV vaccines. TLR agonists alone or mixed with other components are commonly tested adjuvants for therapeutic HPV vaccines (69–72). Recently developed new adjuvants, such as α-Galactosylceramide (α-GalCer), manganese (Mn4^+^)-doped silica nanoparticles (Mn4^+^-SNPs), and very small-size proteoliposomes (VSSP) (73–75), might play roles in reinforcing the efficacy of therapeutic HPV vaccines.

Prophylactic HPV vaccines are mainly protein-based VLPs. Therapeutic HPV vaccines are primarily based on nucleic acid, bacterial/viral vector, protein/peptide or whole cells (76). To date, no therapeutic HPV vaccine has been licensed. Safety profiles and vaccine efficacy for therapeutic HPV vaccines tested in clinical trials are summarized in **Table 3**. A complete regression of lesions triggered by therapeutic HPV vaccines has been reported, with a regression rate ranging from 17.4% to 89.4% in female patients (54, 78, 81, 82, 84–86, 88, 89). The clearance of HPV DNA in infected cells is considered a predictor of vaccine efficacy, as the presence of HPV DNA at the cervical site is often associated with histologic and cytological changes in cervical intraepithelial neoplasia (CIN) (77, 79–81, 83, 87, 90).

**Table 3:** Summary of therapeutic HPV vaccines evaluated as monotherapy in clinical trials.

Name of Vaccine	Sponsor	Adjuvant	Antigens	Indications	Outcomes	Vaccine Types	Phase	Ref.
pNGVL4a-CRT/E7	Sidney Kimmel Comprehensive Cancer Center at Johns Hopkins	N. A.	E7	HPV-16 Positive CIN 2/3	Well-tolerated. Robust immune response.	DNA	I	(77)
AMV002	Jingang Medicine (Australia) Pty Ltd	N. A.	E6, E7	HPV-associated OPSCC	Well tolerated. E6/E7 Specific cellular responses were elicited in 10 of 12 (83.3%) subjects	DNA	I	(78)
INO-3112	Inovio Pharmaceuticals	N. A.	E6, E7	Cervical cancer	Antibody responses were detected in up to 60% of patients.	DNA	II	(79)
VB10.16	Nykode Therapeutics AS	N. A.	E6, E7	CIN2/3	Eliciting CD8^+^ T cells and robust immune responses to regress the lesion size and grade in CIN2/3 patients.	DNA	II	(80)
GX-188E	Genexine	N. A.	E6, E7	CIN2/3	Signicant HPV clearance and histopathologic regression.	DNA	II	(81)
VGX-3100	Inovio Pharmaceuticals	N. A.	E6, E7	CIN	Showed efficacy against CIN2/3 associated with HPV-16 and HPV-18.	DNA	IIb	(82)
ISA 201	ISA Pharmaceuticals	N. A.	E6	HPV positive Tumors or Malignant Lesions	Well tolerated. Robust HPV-16-specific T-cell immunity.	Peptide	I	(83)
PepCan	University of Arkansas for Medical Sciences	Candida skin test reagent	E6	CIN2/3	Regression rate: 50% (50 and 100 μg). A trend of lower doses (50 and 100μg) being more effective than the higher doses (250 and 500μg).	Peptide	I	(84)
MVA E2 vaccine	National Autonomous University of México	N. A.	E2	HPV infection	Histology complete elimination of lesions: 89.3% (female) and 100% (male). HPV DNA clearance: 83%.	Viral vector	III	(85)
ADXS11–001	Advaxis. Inc	N. A.	E7	Recurrent cervical cancer	12-month survival: 30.9%. 18-month survival: 23.6%. ORR: 17.4%.	Viral vector	II	(54)
Tipapkinogen Sovacivec	Taiho Oncology, Inc	N. A.	E6,E7	CIN2/3	CR:24% PR:12%	Viral vector	II	(86)
Vvax001	University Medical Center Groningen	N. A.	E7, E6	CIN 2/3 Cervical Cancer	Well tolerated; Elicited CD4^+^ and CD8^+^ T cell responses against E6 and E7 in all participants.	Viral vector	I	(87)
BLS-M07	BioLeaders	N. A	E7	CIN2/3	CIN 3 cured in 75% patients.	Bacterial vector	I/IIa	(88)
IGMKK16E7	GLOVACC Co Ltd	N. A	E7	CIN2/3	31.7% CR in high-dose recipients E7–specific interferon-γ producing cells increased with level of response (SD, PR, CR)	Bacterial vector	I/II	(89)
NZ8123- HPV16-optiE6	Department of Parasitology, Pasteur Institute of Iran	N.A.	E6	HPV-16 infection	Well tolerated. Long-term and favorable immune responses	Recombinant protein	I	(90)

CIN, Cervical Intraepithelial Neoplasia; OPSCC, Oropharyngeal Squamous Cell Carcinoma. CR, Complete Response; PR, Partial Response; SD, Stable Disease.

In addition, vaccine antigen-specific T-cell responses were comprehensively evaluated. In a Phase I trial, AMV002 vaccination induced E6 and/or E7 specific cellular immune response in 83.3% subjects (78). In a Phase II study of, GX-188E, 67.3% of patients showed histopathologic regression (<CIN1) and 55.8% of patients with viral clearance 36 weeks after GX-188E treatment. Importantly, E6 and E7-specific IFN-γ positive T-cell response in the patients with HPV clearance presented significant increases compared with patients without clearance (81). In a Phase I study of Candida Skin Test, a colorless extract of Candida albicans FDA-approved for use as a human adjuvant, vaccine-induced cellular immunity against HPV-16 E6 was detected in 65% of vaccine recipients. The percentages of Th1 cells and Th2 cells increased significantly, while no change was observed regarding the percentage of Tregs after two doses of vaccine administration.

Several therapeutic HPV vaccines in clinical trials indicated a correlation between vaccine efficacy and vaccine-induced cellular immune responses. The results of Phase I/II of IGMKK16E7, the first oral immunotherapeutic vaccine, showed the number of HPV-16 E7–specific interferon-γ producing cells in blood increased with response levels (stable disease, partial, and complete responses) (89). In a Phase IIb trial of VGX-3100, vaccine-induced T-cell responses to E6 were positively correlated with clinical outcomes rather than E7, while higher frequencies of HPV-specific CD8^+^ CD137^+^ T cells indicated better outcomes (82). Similarly, a recombinant MVA E2 vaccine induced antigen-specific cytotoxic T-cell responses among all vaccinated patients in a Phase III study (85). Accordingly, 90% of female and 100% of male participants exhibited complete regression of lesions 14 weeks after vaccination (85).

## 4 Future directions for HPV vaccine development

### 4.1 Alternative antigenic targets

L1 protein is a commonly used vaccine target for prophylactic HPV vaccines (91). Preliminary data indicated that L1 protein triggers both humoral and cellular immune responses (92, 93). Indeed, L1 capsomeres induced tumor regression in mice via activation of antigen-specific cytotoxic T lymphocytes (94). In HPV-positive oropharyngeal cancer (OPC) patients, E6, E7, and L1 capsid proteins were recognized by HPV-specific CD8^+^ and CD4^+^ T cells (95). However, L1-based HPV vaccines failed to be effective treatment approaches due to the lack of constitutive expression in premalignant and invasive lesions (58). Currently, many therapeutic vaccines in clinical trials primarily target E6 and E7 proteins (96). Alternative antigenic targets, such as E2 and L2, are also in clinical tests (**Table 4**).

**Table 4:** Summary of adjuvants evaluated in preclinical studies of HPV vaccines.

Adjuvant	Vaccine Types	Profiles of Immune Responses with the Addition of Adjuvant	Ref.
NLX/Alum	Protein	Enhanced humoral immune responses, lymphocyte proliferation and Th1 and Th17 responses.	(97)
AS04	Protein	Optimal activation of APCs, which further increases the activation of antigen-specific T cells.	(25)
R-LPS	Protein	Enhanced antibody responses and Th1 immune responses.	(98)
VSSP	Protein	Enhanced anti-tumor responses.	(75)
PCEP	Protein	Rapid seropositivity, dose-sparing, longer-lasting humoral responses.	(99)
CIA06B	Protein	Enhanced splenic cytokine production and activation of memory B cells; longer-lasting humoral responses.	(100)
c-di-AMP/Alum	Protein	Effective Th1/Th2 and cytotoxic immune responses.	(101)
BECC-derived TLR4 agonists/Alum	Protein	Longer-lasting humoral immunity.	(70)
CpG + o/w emulsion	Peptide	Strong CTL responses and tumor eradication.	(71)
BHSSC	Peptide	Improved effector and memory T-cell responses; inhibition of HPV-related tumors growth.	(102)
Candida skin test reagent (Extract of Candida albicans)	Peptide	Significant polarization of Th1 responses.	(103)
Mn4^+^-SNPs	Peptide	DC maturation; cytosolic delivery of antigens; antigen-specific CD8^+^ T cell responses; remission of tumor.	(74)
CpG and Poly I:C	Peptide	Enhanced antigen-specific cellular immune responses; abolished tumor growth.	(104)
S-540956 (a CpG Oligonucleotide)	Peptide	Activation of plasmacytoid dendritic cells (pDCs); induction of CD8^+^ T cell responses via TLR9 in a CD4^+^ T cell-independent manner; remission of tumor.	(72)
Beclin-1	DNA	Increased production of IFN-γ and highly inhibited tumor progression.	(105)
MPL+α-GalCer	DNA	Increased lymphocyte proliferation, CTL activity, cytokine responses, and tumor remission.	(73)
mRNA-encoded STING^V155M^	mRNA	Enhanced antigen-specific T cell responses by activating type I IFN responses via the nuclear factor kB (NF-kB) and IFN-stimulated response element (ISRE) pathways; reduced HPV^+^ tumor growth.	(106)
Chitosan	DNA vaccine	Strong induction of E7-specific CD8^+^ T cells, IFN-γ responses, and therapeutic antitumor effects	(107)

HPV DNA integration and diminished E2 expression were essential for the oncogenic transition of HPV-infected cells (108). E2 controlled HPV DNA transcription and suppressed E6 and E7 expression (96). As a result, E2 was probably irrelevant to the therapeutic vaccine target. However, E2-specific T-cell responses were related to the lack of progression into high-grade intraepithelial lesions (HSILs) (60). Accordingly, more potent E2-specific T-cell responses have been reported in patients with resolved cervical dysplasia, supporting the role of E2 as a candidate for vaccine antigens in immunotherapy of pre-cancerous cervical lesions (109). E1 is continuously expressed in the basal epithelial cells after HPV infection and plays an important role in virus replication (110). E1 is crucial for carcinogenesis (111). However, E1 might not be an ideal target for vaccine antigens due to its ubiquitination and rapid degradation attributes (112, 113).

The minor capsid protein L2 is a promising candidate for an HPV vaccine since the linear neutralizing epitopes on the L2 N-terminus are well conserved across several HPV genotypes (114). In mouse models, HPV-16 L2 antigen-induced neutralizing antibodies protected against infectious HPV-16/45 challenges (115, 116). However, L2 might be less immunogenic since it is unable to form VLPs (117). Therefore, strategies to enhance the immunogenicity of L2 protein have become attractive (114, 118, 119). Notably, peptide antigens (120, 121) and HSP70-based fusion proteins are widely tested in both preclinical and clinical studies (122, 123).

### 4.2 Adjuvant optimization

Cervarix^®^ and Gardasil^®^ were formulated with AS04 and amorphous aluminum hydroxyphosphate sulfate, respectively (124, 125). Alum-based adjuvants could induce Th2-biased immune responses (126). The immunostimulant MPL, one component of the AS04 adjuvant, activates innate immune responses through Toll-like receptor 4 (TLR4) and induces a mixed Th1/Th2 immune response (127). Participants received Cervarix^®^ displayed stronger humoral and CD4^+^ T cell responses against HPV-16 and 18 than patients received Gardasil^®^ (125, 128, 129). Meanwhile, distinctive immune profiles induced by HPV vaccines formulated with an Alum-based adjuvant, or AS04, have been reported (129, 130).

Adjuvants may affect the strength and durability of antigen-specific immune responses. Exploratory studies of novel adjuvants used alone or in combination have highlighted their potential use in HPV vaccines (**Table 4**). Bacterial-derived components, such as Beclin-1 (105), AS04 (NLX+Alum) (25), CIA06B (nontoxic derivative of lipopolysaccharide (CIA05) + Alum) (100), rough LPS (R-LPS) (98), and bacterial enzymatic combinatorial chemistry (BECC)-derived TLR4 agonists/Alum (70), are widely utilized in exploratory studies.

In addition, novel molecules with distinctive contributions to immunogenicity are being evaluated in preclinical studies of HPV vaccines, such as CpG oligodeoxynucleotide (CpG) (71, 104), polyinosinic-polycytidylic acid (Poly I:C) (104), α-Galactosylceramide (α-GalCer) (73), very small size proteoliposomes (VSSP) (75), poly di (carboxylatoethylphenoxy) phosphazene (PCEP) (99), c-di-AMP (101), S-540956 (a CpG Oligonucleotide) (72), the metal ions like manganese (Mn4^+^)-doped silica nanoparticles (Mn4^+^-SNPs) (74), plant extracts like Bai Hua She She Cao (BHSSC) (102), and mRNA-encoded STING^V155M^ (106), Candida skin test reagent (Extract of Candida albicans) (103).

Although many novel adjuvants have demonstrated their unique efficacy in modulating immune responses in mouse models, only a few have been licensed for use in humans to date, primarily due to safety concerns. Therefore, novel HPV vaccine adjuvants require further safety and efficacy validation in clinical trials.

### 4.3 Combination with immune checkpoint blockade therapy

HPVs employ multiple strategies to evade the host’s immune response, including interference with antigen presentation, downregulation of antigen production, inhibition of antiviral molecules, suppression of type 1 T helper (Th1) cell immune responses, and promotion of regulatory T cell (Treg) responses (84, 131). Therapeutic HPV vaccine-evoked antigen-specific T cells are the key to tumor cell elimination and rely primarily on the loss of inhibitory signals in the tumor microenvironment (TME). Thus, the combination of therapeutic HPV vaccines and immune checkpoint inhibitors may show synergistic effects in tumor regression.

Preclinical studies have demonstrated that therapeutic HPV vaccines, such as adenovirus delivery vectors carrying modified HPV-16 E6 and E7 genes, HPV-16 E7 long peptide with DCs, and the Lm-LLO fused-E6 vaccine (Lm-LLO-E6), significantly promoted tumor regression and survival rate in tumor-bearing mouse models when used with a programmed death-ligand 1 (PD-L1) inhibitor (132–134). The clinical trials of therapeutic HPV vaccines combined with ICB therapeutic modalities are mainly in Phase I/II. Preliminary data show good efficacy and safety, and further verification in later clinical trials is needed. The current status of combination trials is listed in **Table 5**.

**Table 5:** Clinical trials of therapeutic HPV vaccines in combination with ICB therapy.

Vaccine	Combination	Sponsor	Phase (N)	Safety Profiles	Efficacy	Ref.
GX-188E	Pembrolizumab	Genexine, Inc.	II (36)	AEs: 44%; Grade 3–4 TRAE: 11%. No treatment-related deaths.	ORR: 42%. CR: 15%. PR: 27%.	(135)
ISA 101	Nivolumab	ISA Pharmaceuticals	II (24)	Grades 3–4 toxicity: 2 patients; Asymptomatic grade 3 transaminase level elevation: 1 patient; Grade 4 lipase elevation: 1 patient.	mPFS: 2.66 months. mOS: 15.3 months. 2-year OS rate: 33%. objective response: 38% (without progression at 3 years)	(136)
TG4001	Avelumab	Transgene	Ib/II (34)	N. A.	ORR: 23.5%. CR: 2.9%. PR: 20.6%.	(137)
PRGN-2009	M7824	National Cancer Institute (NCI)	I/II (6)	Grade 1–2 flu-like syndrome; Injection site reactions, fatigue, and rash.	SD: 2/3 patients. Increased T cells were detected in all patients, with 3/6 (50%) developing HPV-16 T cells and 5/6 (83%) developing HPV-18 T cells.	(138)
PDS 0101	M9241 and M7824	PDS Biotechnology Corporation	II (14)	Manageable safety profile.	Evidence of notable clinical activity for patients with both checkpoint naïve and refractory HPV-16+ advanced malignancies. ORR: 10/14 (71%). CR: 1(anal cancer). PR: 9 (3 cervical, 2 vulvar/vaginal, 2 anal, 2 oropharyngeal).	(139)
MG1-E6E7	Atezolizumab	Turnstone Biologics, Corp	I (75)	N. A.	N. A.	NCT03618953
ISA101b	Cemiplimab	ISA Pharmaceuticals	II (194)	N. A.	N. A.	NCT03669718
INO-3112	Durvalumab	M.D. Anderson Cancer Center	II (77)	N. A.	N. A.	NCT03439085

AE, Adverse Event; TRAE, Adverse Event; ORR, Objective Response Rate; CR, Complete Response; PR, Partial Response; SD, Stable Disease; OS, Overall Survival; DCR, Disease Control Rate; N.A., not available.

### 4.4 HPV L1 VLP vaccine as adjuvant therapy

L1 VLPs-based HPV vaccines have been approved primarily for the prevention of cervical cancer in populations without preexisting HPV infection. Studies have suggested an indispensable role of HPV L1 VLP vaccines as adjuvant therapy after surgical treatment for patients suffering from HPV-related clinical diseases such as recurrent respiratory papillomatosis (RRP) (140) and cervical intraepithelial neoplasia (CIN) (141). The data reported that surgical intervention and HPV vaccination combination therapy can effectively reduce the recurrence and severity of respiratory papillomatosis (142). Likewise, another study has reported that HPV vaccination significantly prolongs the intervals between surgical procedures and reduces the number of procedures in the majority of RRP patients (143). Most CINs are caused by HPV infection (144) and might progress to cervical cancer (145). CIN 1 rarely progresses to malignancy with a high potential for regression. Instead, CIN 2 and 3, as the high-grade lesions, become highly cancerous with a lower potential for regression (146, 147). Studies have reported that vaccination with the quadrivalent HPV vaccine in patients after loop electrosurgical excision procedure (LEEP) treatment significantly prevents the recurrence of CIN2–3 related to vaccine HPV types compared with non-HPV-vaccinated patients (148). These data demonstrate that HPV vaccination plays a critical role as adjuvant therapy in HPV-related disorders other than cancer.

### 4.5 Exploration in formulation strategy, dosing regimens, and expansion of vaccine recipients

#### 4.5.1 Alternative strategies for HPV vaccine formulation

High cost, cold chain storage and delivery, and requirements for trained vaccinators limit vaccination coverage, which prompts the development of nano- or micro-particulate vaccines (149). Nanotechnology has been extensively used in vaccine development via the utility of advanced nano-based particles or materials designed for antigen delivery or as adjuvants to enhance immune responses (150). Archaeosomes are commonly used in cancer vaccines as delivery tools. HPV DNA vaccines containing the truncated L1, E6, and E7 genes in combination with archaeosomes induce strong cytolytic immune responses to eliminate tumor cells (151). Gold nanoparticles can also be potentially used in the HPV vaccine to strengthen cytotoxic immune responses by increasing oxidative stress (152). Chitosan is also used as a nanoparticle adjuvant due to its non-toxic, highly biocompatible, low susceptibility, and biodegradation properties (153). Chitosan-based DNA vaccines expressing HPV-16 E7 significantly promote T cell-mediated immune response and antibody production against HPV-induced tumors (154). Hence, the nanotechnology-created HPV vaccine would provide more options for the treatment of HPV-related diseases. Additionally, HPV protein antigens can be formulated into glassy microspheres using spray-dried techniques and then coated by atomic layer deposition (ALD) with nanometer-thin protective layers of alumina to improve their thermostability (155). Given the advantage of nanotechnology utilization in HPV vaccine formulation, it would provide more options for patients in prevention or treatment of HPV-related diseases. However, the current nanoparticle agents for the HPV vaccine are still far away for human use, with uncertainty concerning. Further studies regarding the safety, stability, potency, and rapid production of HPV are required to be explored.

Oral administration of the HPV vaccine is easy, with favorable safety profiles and potential long-lasting protective effects in the intestine (156). Oral vaccination could diminish vaccine hesitancy and boost vaccine coverage (157). A clinical study has reported that an oral therapeutic vaccine has demonstrated safety and potency to induce persistent immunity (90). The combinational application of a nanotechnology-based DNA vaccine and a VLP vaccine could potently stimulate both humoral and cellular immunity, which has been investigated for HIV infection (158). Oral films have been extensively used in formulations for the administration of many drugs (159). Film-based HPV vaccines would be attractive as a novel type of HPV vaccine to treat HPV-related carcinogenesis. Film materials make the administration of the HPV vaccine easy and reduce the risk of choking or suffocation in pediatric, geriatric, and psychiatric patients (160). The film dosage forms incorporated with DNA- and VLP-based vaccines offer a promising approach for future HPV development. While intramuscular injection of HPV vaccines has shown clinical effectiveness, the efficacy of orally administered HPV vaccines remains uncertain. Additional research is needed to investigate the bioavailability, immune response, and safety aspects of orally administered HPV vaccines in preclinical studies and clinical trials.

#### 4.5.2 Dosing regimens

For novel HPV vaccines, reducing the vaccination frequency would be a wise strategy. To achieve this, participants will be administered higher concentrations of HPV antigens. A recent study demonstrated the efficacy of a single dose of the HPV vaccine in young women in Africa (161). Another report has shown one dose of quadrivalent HPV vaccination had comparable effectiveness as two or three doses in preventing cervical intraepithelial neoplasia (162). More studies to compare the efficacy among the one-dose, two-dose, and three-dose regimens are presented in **Table 6**. This information indicated that the potency induced by one dose vaccination was almost similar to that of two or three doses in preventing HPV infection. Additionally, self-boosting vaccination for HPV could reduce the dosing frequency and improve its lasting effectiveness by loading HPV antigens into microparticles. This could be released over time, continuously boosting its immune response (167, 168).

**Table 6:** A summary of one-dose regimen for HPV vaccines.

Location	Participants	Vaccines	Follow-up	Outcomes	References
Tanzania	Schoolgirls aged 9–14 years	Cervarix/ Gardasil-9	1 year	HPV 16 IgG positive: 99% (one dose) vs 100% (two doses) vs 100% (three doses)	(163)
USA	Females aged 9 to 26 years	Gardasil	1 year	Hazard ratio for histologically confirmed preinvasive cervical disease: 0.64 (one dose) vs 0.72 (two doses) vs 0.66 (three doses)	(164)
India	Girls aged 10–18 years	Gardasil	9 years	Vaccine efficacy against HPV 16/18 infection: 95.4% (one dose) vs 93.1% (two doses) vs 93.3% (three doses)	(165)
Costa Rica	Women aged 18 to 25 years	Cervarix	11.3 years	Vaccine efficacy against HPV16 or 18 infection: 82.1% (one dose) vs 83.8% (two doses) vs 80.2% (three doses)	(166)

The current HPV dose schedule recommended by the WHO Strategic Advisory Group of Experts on Immunization (SAGE) is as follows: 1) One or two-dose schedule for the primary target of girls aged 9–14 years; 2) One or two-dose schedule for young women aged 15–20 years; 3) Two doses with a 6-month interval for women older than 21 years (169). Some experts suggest that single-dose vaccination is less expensive, supports more compliance, and is therefore logistically easier compared with multiple doses, making it more feasible to vaccinate more women in low- and middle-income countries (170). Similarly, the Joint Committee on Vaccination and Immunization (JCVI) statement also considers the potential change to a one-dose schedule for the routine HPV immunization program (171).

#### 4.5.3 Expansion of vaccine recipients

In 2018, the FDA approved the expanded use of Gardasil 9^®^ to include individuals 27 through 45 years old (172). According to the WHO guidelines, HPV vaccination is usually recommended for boys and girls aged 11 to 12, but can be given as early as age 9 years for the first dose before sexual contact and exposure to HPV. Children between the ages of 11 to 12 years are suggested to get 2 doses of the HPV vaccine 6 to 12 months apart. For teens and young adults at ages 15 through 26, they need three doses of the HPV vaccine. Early prevention with the HPV vaccine is a safe and effective way to reduce HPV infection (173). Studies have demonstrated that adolescents who initiated the HPV vaccine series at age 9 or 10 were 22 times more likely to complete the two-dose series by age 15 than those who started the series at age 11 or 12 (174). To date, the safety and efficacy of the HPV vaccine, for example, Gardasil^®^, in children below 9 years of age have not been established. Thus, whether HPV vaccination programs are adopted for children below 9 years old requires further investigation. A quality improvement initiative performed in the Nationwide Children’s Hospital system utilizing electronic medical record alerts has shown rapid uptake of the HPV vaccine before age 11, suggesting a willingness by parents and providers to initiate the vaccine earlier than previously recommended (175). On the other hand, parents have worried that early HPV vaccination in adolescents would increase sexual behavior, thereby increasing the risks of HPV infection. However, a study reported that HPV vaccination has not increased sexual activity or accelerated sexual debut in a college-aged cohort of men and women (176). Experts believe that early HPV vaccination against HPV can not only increase completion rates but also reduce cancer mortality.

HPV vaccination has been expanded to young males since 2009 in the United States (177), and gender-neutral (GN) HPV vaccination has been adopted in 33 out of 107 countries as of 2019, with 4% of males worldwide get vaccinated (178). Data from a community randomized clinical trial (NCT000534638) indicated the advantage of GN over girls-only (GO) vaccination against HPV infection (179). Vaccination in both genders might help to build resilient cervical cancer prevention (180). Despite preliminary scientific evidences and potential public health benefits, more issues should take into consideration regarding the deployment of HPV vaccination program in the future. Due to limited vaccine supply, the WHO recommended to postpone the GN vaccination policy in 2019 (181). Factors affecting HPV vaccination in males have been reviewed by several reports, including limited economic resources, as well as ethical and legal considerations (177, 182, 183).

## 5 Future perspective

Prophylactic HPV vaccines have shown efficacy in preventing HPV transmission and infection, controlling the incidence of HPV-related cancers and genital warts, and improving clinical manifestations. Correlates of protection need to be better defined, such as the magnitude of humoral and cellular immune responses, specific antibody characteristics, adjuvants, and age of recipients. This information might guide the development and deployment of HPV vaccines for protection against HPV infection. Enhancing vaccine efficacy through diverse L1 antigens, potent adjuvants for cellular immunity, and therapeutic HPV vaccines targeting E6 and E7 proteins, particularly in conjunction with immune checkpoint blockade, may pave the way for eradicating HPV infections and associated cancers. As an attractive research area, therapeutic HPV vaccines in clinical trials have shown promising results in safety, HPV DNA clearance, tumor regression, and antigen-specific T-cell responses. The investigation is constantly progressing toward enlarging the protective breadth of prophylactic vaccines, with most already in the late stages of clinical trials. Promising preclinical findings support novel antigenic targets and adjuvants for vaccine design. However, confirming these findings in clinical trials is necessary. Moreover, recent progress in the exploration of vaccine formulation, immunization schedules, and age expansion may help reduce HPV transmission and infection due to increased vaccine coverage.

## Author contributions

LX: Writing – review & editing, Supervision, Funding acquisition, Conceptualization. RW: Writing – review & editing, Writing – original draft. HH: Writing – original draft. CY: Writing – original draft. XL: Writing – review & editing. YW: Writing – review & editing.
